# Antagonistic Effects of Tetramethylpyrazine on Hypoxic Respiratory Depression in Rats

**DOI:** 10.1155/2020/6456017

**Published:** 2020-09-29

**Authors:** Li Li, Bo Gao, Meifang Liu

**Affiliations:** School of Pharmacy, Jining Medical University, Rizhao 276826, China

## Abstract

**Objective:**

Tetramethylpyrazine (TMP) is an alkaloid extracted from the root and stem of the traditional Chinese herbal medicine called Chuanxiong. The present study aims to study the effects of TMP on hypoxic respiratory depression in rats. *Materials and methods*. The effects of TMP on respiratory responses of rats induced by hypoxia were observed by diaphragm electromyogram (EMG) recording. The effects of TMP on the protein expression of FOS and acid sensing ion channel_1a_ (ASIC_1a_) in the brainstem induced by hypoxia were investigated by immunohistochemistry.

**Results:**

The respiration of rats was first excited and then depressed during hypoxia treatment, while TMP pretreatment could significantly antagonize the respiratory depression induced by hypoxia (*P* < 0.01). Hypoxia obviously induced the protein expression of FOS (*P* < 0.01) and ASIC_1a_(*P* < 0.05) in the brainstem, which can be also significantly inhibited by TMP pretreatment.

**Conclusions:**

TMP has protective effects on hypoxic respiratory depression, and the mechanisms might be concerned with its downregulation of FOS and ASIC_1a_ in the brainstem induced by hypoxia.

## 1. Introduction

It is known that the essential respiratory centers are located in the lower brainstem, which plays an important role in producing respiratory rhythm and regulating respiratory movement. Hypoxia is a common pathological process, which can injure the neurons in the respiratory centers of the brainstem and lead to respiratory depression and even death. Therefore, it is of profound significance to find some medicine to antagonize respiratory depression caused by hypoxia. Tetramethylpyrazine (TMP), also called ligustrazine, is an alkaloid extracted from the root and stem of the traditional Chinese herbal medicine called Chuanxiong. It is reported that TMP has multiple pharmacological actions, including dilating blood vessel, increasing artery blood flow, suppressing the activation, and aggregation of blood platelets. And TMP has been used as clinic treatment for various hypoxic/ischemic diseases in China [1]. However, little is known about the role of TMP in hypoxic respiratory depression and the exact mechanisms.

Pharmacokinetic study showed that TMP could pass through the blood-brain barrier and reach a steady-state concentration in the brainstem for a long time [2]. This study suggested that the brainstem might be a critical target for the protective effects of TMP against hypoxia-induced injury. Since brain tissues are highly sensitive to hypoxia and TMP may have a protective effect on neurons, we inferred that TMP may attenuate the hypoxic injury on neurons in the respiratory center of the brainstem and thus antagonize the respiratory depression induced by hypoxia.

Large amount of genes were induced by acute hypoxia, such as FOS, hypoxia inducible factor (HIF), nitric oxide synthase (NOS), and so on [3]. FOS is a kind of immediate early gene, which is upregulated by hypoxia in a very short time [4], and it is responsible for the transcription of some late-response genes and cell damage. It is considered that the degree of cell damage is positively correlated to the expression level of FOS [5]. Acute hypoxia is also accompanied with the decrease of pH in the extracellular fluid due to the glycolysis enhancement. Acid sensing ion channel_1a_ (ASIC_1a_) is a subtype of ASIC_S_ [6] and can be activated when the pH of extracellular fluid just reaches 6.9 [7]. Escoubas et al. reported that the expression of ASIC_1a_ increased after hypoxia and pretreated with pcTX_1_, a specific blocker of ASIC_1a_, could attenuate neuronal damage caused by hypoxia in brain slice preparation [8]. Thus, FOS and ASIC_1a_ might be involved in the protective effects of TMP on hypoxic respiratory depression.

In the present study, we elucidated the effects of TMP on hypoxia-induced respiratory depression in rats. In addition, we further investigated the influences of TMP on the protein expression of FOS and ASIC_1a_ induced by hypoxia to explore the underlying protective mechanisms.

## 2. Materials and Methods

### 2.1. Animal Welfare and Ethical Statements

Sprague-Dawley (SD) rats of either gender, weighed 250–300 g, were used in this study. All experimental procedures complied with the recommendations of ARRIVE (Animal Research: Reporting of In Vivo Experiments) guidelines [9]. The Institutional Animal Care and Use Committee of Jining Medical University approved the procedures. Rats were housed in a pathogen-free environment and allowed to acclimate to the environment for 7 d before inclusion in an experiment.

### 2.2. Establishment and Treatment of the Rat Hypoxia Model

**Figure 1:**
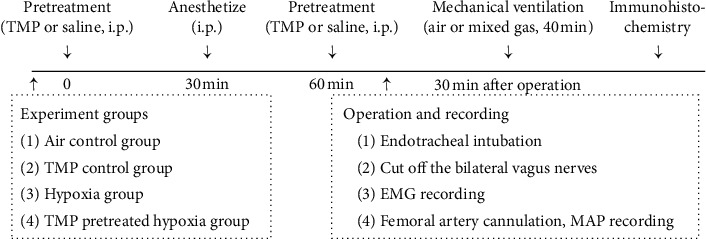
A schematic diagram depicted the experimental design in the present study. EMG, electromyogram of diaphragm; MAP, mean arterial pressure.

The schematic diagram (Figure 1) depicted the schedules of the animal experiments. 32 SD rats were divided randomly into 4 groups, including the air control group, TMP control group, hypoxia group, and TMP pretreated hypoxia group. Each group contained 8 animals. All animals were anesthetized via the intraperitoneal (i.p.) injection of phenobarbital sodium (30 g/L) before operation. Endotracheal intubation was performed, and bilateral vagal nerves in the neck were cut. The femoral artery was cannulated for mean arterial pressure (MAP) measurement, and rectal temperature was kept at 37°C ± 0.4°C. Diaphragm electromyogram (EMG) was recorded to detect the respiratory indexes. TMP used in this study was provided by Shanghai First Pharmaceutical Company (Shanghai, China, batch number ST8120), and the purity of TMP is higher than 98% (HPLC). In the TMP control group and TMP pretreated hypoxia group, rats were pretreated with TMP (80 mg/kg, i.p.) 30 min before and after anesthesia, the interval between the two injections is 1 h, and rats in the air control group and hypoxia group received saline injections (i.p.). After the recovery time of 30 min, rats in the hypoxia group and TMP pretreated hypoxia group were ventilated with mixed gas (including 8% O_2_ and 92% N_2_) at the flow rate 1300 ml/min via the side port of the tracheal cannula to establish the acute hypoxia model; rats in the air control group and TMP control group were ventilated with air at the same flow rate. According to the preliminary experimental results, the hypoxia duration was set at 40 min, and then, animals were sacrificed and perfused with 4% paraformaldehyde solution transcardially for immunohistochemical staining.

### 2.3. Monitoring of the Respiratory Indexes and MAP

The signal of diaphragm EMG and MAP were amplified by the multichannel physiological recorder (RM-6000, Kohden, Japan) and recorded by the PowerLab biological signal recording and analysis system (ML-500, AD Instruments, Australia). Inspiratory duration (TI), expiratory duration (TE), respiratory frequency (RF), and inspiratory amplitude (Amp) were taken as indexes to observe the changes of respiration. Diaphragm EMG was recorded continuously; the mean values of TI, TE, RF, and Amp were measured every minute. According to the manifestation of respiration in the hypoxia group, breathing is depressed 40 min after hypoxia; however, the TMP pretreated hypoxia group is not depressed, so we take 1 min, 10 min, 20 min, 30 min, and 40 min as our observation time points and calculate the changing rates of the above indexes. *∆ x*% = (observed value− prehypoxic control value)/prehypoxic control value × 100％.

### 2.4. Immunohistochemical Staining

All the animals were performed left ventricular perfusion after ventilation for 40 min. 250 ml normal saline and PFA solution (4% paraformaldehyde in 0.1 M phosphate buffer, pH = 7.4) were successively used to perfuse and fix the brain tissue. The brainstem were removed and postfixed in PFA solution overnight and then cryoprotected in 20% sucrose at 4°C. After liquid nitrogen freezing, each brainstem was sectioned successively from caudal end of the spinal cord to the head end of the pons on a freezing microtome, and the sections were 30 *μ*m thick. Two successive sections were taken per 6 sections for the immunohistochemical staining. The immunostaining of FOS and ASIC_1a_ in the brainstem was performed using a standard streptavidin-peroxidase (Sp) kit. The primary antibodies and other related reagents were all bought from American Santa Cruz Company (US). The speciﬁcity of antibodies was validated by positive and negative tests. The experimental operation procedure is according to the instruction of the immunochemistry kit. Antigen retrieval of all sections was performed with boiling sodium citrate buffer (pH = 6.0). Two sets of sections were incubated with rabbit anti-FOS (1 : 50) or rabbit anti-ASIC_1a_ antibody (1 : 100) at 4°C overnight, separately. After the immunohistochemical staining, all sections were subjected to the dehydration procedure and sealed for microscopy observation.

### 2.5. Analysis of Tissue Section Staining

FOS- and ASIC_1a_-positive nuclei in the brainstem were observed under the microscope. The gray values and the number of positive neurons in the nuclei of the brainstem were measured by a computer-assisted image analyzer (HPIAS-1000 high precise color image measure system, Tongji Qianping Image Engineer Company, China). The gray value reflects the intensity of an antigen in the nuclei of the brainstem; the higher the gray value, the lower the intensity of the target antigen. The gray value was calculated by randomly selecting 10 fields of each nucleus in each section. The mean gray value of all the chosen fields was the gray value of the neural nucleus of this animal. The number of positive neurons in the corresponding nucleus of one animal was determined by the mean value of the number of positive neurons of all the taken sections.

### 2.6. Statistical Analysis

Data were expressed as means ± SD. Statistical analysis was performed using IBM SPSS Statistics 22.0 software and Prism 8.0. The results were analyzed by *t*-test or one-way/two-way analysis of variance followed by a Bonferroni post hoc test for multiple comparisons, and *P* < 0.05 was considered statistically significant.

## 3. Results

### 3.1. Effects of TMP on the Changes of Respiration Induced by Hypoxia

**Figure 2:**
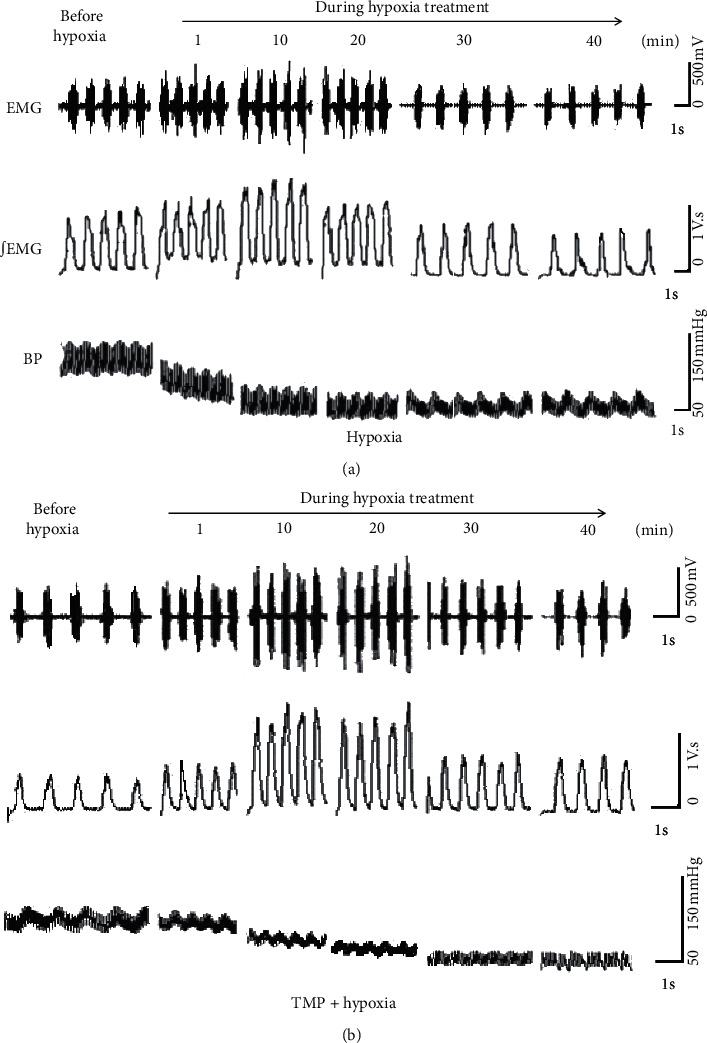
Effects of TMP on the changes of respiration and blood pressure induced by hypoxia. (a) The respiration and blood pressure changes of rats in the hypoxia group. (b) The respiration and blood pressure changes of rats in the TMP pretreated hypoxia group. EMG, raw electrogram of diaphragm; ∫EMG, integrated electrogram of diaphragm; BP, blood pressure.

Respiration changes of four groups were recorded. Diaphragm EMG barely changed for rats in either the air control group or the TMP control group (*P* < 0.05), which indicated that both mechanical ventilation and TMP had no obvious effects on respiration (Table S1). In the hypoxia group, respiration was first excited and then depressed during hypoxia treatment, and obviously, respiration depression was observed at 40 min after hypoxia treatment. While, in the TMP pretreated hypoxia group, hypoxia treatment induced respiratory excitation but did not lead to obvious respiratory depression (Figure 2).

**Figure 3:**
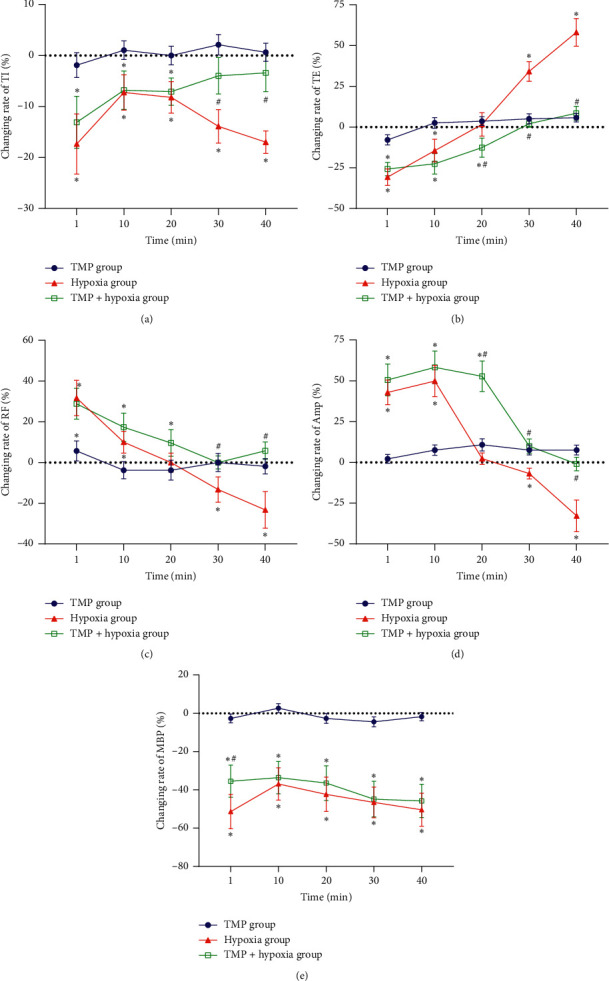
Effects of TMP on the changing rates (%) of TI, TE, RF, Amp, and MBP at various time points after hypoxia treatment. (a) Changing rate of TI. (b) Changing rate of TE. (c) Changing rate of RF. (d) Changing rate of Amp. (e) Changing rate of MBP. ^*∗*^*P* < 0.01*vs*. the TMP control group, ^#^*P* < 0.01*vs*. the hypoxia group. TI, inspiratory duration, TE, expiratory duration, RF, respiratory frequency, Amp, inspiratory amplitude; MBP, mean blood pressure.

Furthermore, four indexes for respiration obviously changed in the hypoxia group and TMP pretreated hypoxia group compared with that before hypoxia treatment (Figure 3, Table S1). In the hypoxia group, TI was shorten at each observation time point after hypoxia treatment (*P* < 0.05), while TE was shorten at 1 min and 10 min, (*P* < 0.05) but extended at 30 min and 40 min after hypoxia treatment (*P* < 0.01); by contrast, RF and Amp were increased at 1 min and 10 min (*P* < 0.05) but decreased at 40 min after hypoxia treatment (*P* < 0.05). In the TMP pretreated hypoxia group, TI (*P* < 0.05) and TE (*P* < 0.05) were evidently decreased, but RF and Amp were increased at 1 min, 10 min, and 20 min after hypoxia treatment (*P* < 0.05). For the above indexes, there was no obvious statistical difference at 30 min and 40 min after hypoxia than prehypoxia (*P* < 0.05). More importantly, at 30 min and 40 min after hypoxia, the reduction rate of TI and the increase rate of TE in the rats of the TMP pretreated hypoxia group were smaller than that of the hypoxia group (*P* < 0.01), and the changing rate of RF and Amp in the rats of the TMP pretreated hypoxia group differed markedly from the hypoxia group (*P* < 0.01). In addition, at 30 min and 40 min after treatment, the changing rates of TI, TE, RF, and Amp showed no discernible difference between the TMP control group and the TMP pretreated hypoxia group (*P* < 0.05). These results demonstrated that TMP pretreatment significantly antagonize the respiratory depression caused by hypoxia (Table S1, Figure 3).

In addition, changes of MAP were also recorded in the present study. The results showed that MAP significantly decreased in both the hypoxia group and the TMP pretreated hypoxia group at all observation time points compared with the TMP control group (*P* < 0.01). And TMP pretreatment only slightly attenuated the MAP reduction at 1 min after hypoxia treatment (*P* < 0.01). The results suggested that the improvement of TMP on respiratory depression is more lasting and potent than that on MAP reduction, and the protective effects of TMP on respiration depression caused by hypoxia are relatively special (Figure 3).

### 3.2. Effects of TMP on the Expression of FOS and ASIC_1a_ in the Neurons of the Brainstem

**Figure 4:**
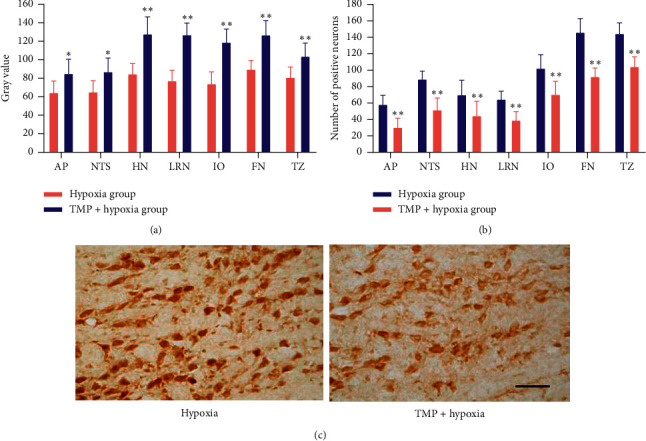
Effects of TMP on the expression of FOS in the nuclei of the brainstem after hypoxia treatment. (a) Gray values of various FOS-positive nuclei. (b) Quantitative analysis of FOS-positive neurons in various FOS-positive nuclei. (c) Photomicrographs of representative TZ stained with FOS antibodies. ^*∗*^*P* < 0.05*vs.* the hypoxia group, ^*∗∗*^*P* < 0.01*vs.* the hypoxia group. Scale bar, 50 *μ*m. AP, area postrema; NTS, nucleus of solitary tract; HN, hypoglossal nucleus; LRN, lateral reticular nucleus, IO, inferior olivary nucleus; FN, facial nucleus; TZ, trapezoid nucleus.

The results from immunohistochemical staining showed that FOS-positive neurons were not detected in the brainstem of either the air control group or TMP control group, and the expression of FOS was extremely induced by hypoxia treatment in various nuclei of the brainstem, including area postrema (AP), nucleus of solitary tract (NTS), hypoglossal nucleus (HN), lateral reticular nucleus (LRN), inferior olivary nucleus (IO), facial nucleus (FN), and trapezoid nucleus (TZ). TMP pretreatment significantly decreased the expression of FOS in all of the above nuclei (Figure 4). Both the gray values and the quantity of FOS-positive neurons showed an obvious difference between the two groups (*P* < 0.05,  *P* < 0.01).

**Figure 5:**
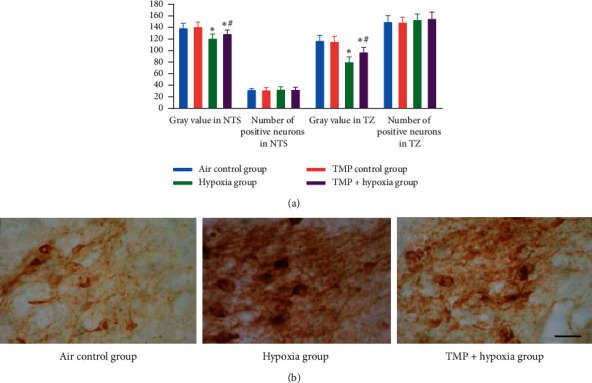
Effects of TMP on the expression of ASIC_1a_ in NTS and TZ after hypoxia treatment. (a) Gray values and quantity of ASIC_1a_-positive neurons in NTS and TZ. (b) Photomicrographs of representative NTS stained with ASIC_1a_ antibodies. ^*∗*^*P* < 0.05*vs.* the air control group, ^#^*P* < 0.01*vs*. the hypoxia group. Scale bar, 50 *μ*m. NTS, nucleus of solitary tract; TZ, trapezoid nucleus.

The results (Figure 5) showed that ASIC_1a_ was detected in both NTS and TZ of the brainstem in each group. The quantity of ASIC_1a_-positive neurons showed no significant differences among the four groups (*P* > 0.05). Hypoxia treatment obviously decreased the gray value of ASIC_1a_ in the two nuclei compared with the air control group (*P* < 0.05), and TMP pretreatment significantly antagonized the alternation of ASIC_1a_(*P* < 0.05).

## 4. Discussion

TMP is an alkaloid extracted from the traditional Chinese herb Chuanxiong, which has multiple pharmacological actions and has been used as clinical treatment for various hypoxic/ischemic diseases in China [1]. However, little is known about the role of TMP on hypoxic respiratory depression and the exact mechanisms. The present study revealed that TMP pretreatment antagonized the respiratory depression induced by hypoxia. It is a known fact that respiration was first excited and then depressed during hypoxia treatment [10]. Mild hypoxia elicits respiratory excitation via peripheral chemoreceptors; however, severe hypoxia induces neuronal damage of respiratory center in the brainstem and leads to respiratory depression [11]. Enhancement of glycolysis, calcium overload, and generation of free radicals are the common mechanisms of hypoxia-induced neuronal damage [12] and generally considered to be involved in the injury of respiratory center in the brainstem by hypoxia. It is reported that TMP can inhibit glycolysis, enhance aerobic metabolism, reduce calcium overload, and promote the scavenging of free radicals [13, 14]. Therefore, all these effects might be involved in the protection of TMP on the neurons of the respiratory center and thus against hypoxic respiratory depression.

FOS is an immediate early gene, and it is rapidly expressed under the induction of such factors as hypoxia, trauma, and epilepsy. FOS expression can be detected by immunohistochemical staining 15 min after stimulation. However, there was no FOS expression in multiple tissues such as the brainstem under normal conditions [15]. Our immunohistochemical study of FOS demonstrated that there was no expression in the rats of the air and TMP control groups in the brainstem, but their expression was significantly increased 40 min after hypoxia in the brainstem of rats, which was consistent with literature reports. FOS and JUN, subunits of the AP-1 transcription factor, can provoke apoptosis by downregulating bcl-2 [16]. According to the studies of recent years, the expression of FOS is positively correlated to the extent of cell damage, and the FOS expression increases with the period and intensity of nociceptive stimulation [17, 18]. Multiple studies showed that Fos expression has been widely used as a sensitive cellular marker for neuronal activation induced by a variety of stimuli, and the FOS-positive neural nuclei is similar to the neural nuclei on the neural pathway gotten by tracer studies [19–21]. In the present study, expression of FOS in multiple neural nuclei after hypoxia indicated that hypoxia led to neuronal damage. Meanwhile, it may also shed light on the complicated projections between the FOS-positive neural nuclei.

NTS belongs to the dorsal respiratory group, and it is a transfer station where the signals of the respiratory center output and peripheral chemoreceptor signal input [22]. NTS is involved in breathing regulation by extensive connections with other neural nucleus in the brainstem. Zoccal et al. showed that electrical discharge of the 90% inspiratory units in NTS decreased after hypoxia, but 80% expiratory units increased [23]. Thus, the changes of discharge activities of NTS could be one of the reasons for respiratory depression. Huangfu et al. showed that the discharge activities of respiratory neurons were first excited and then suppressed after hypoxia in medulla brain slices [24]. This study indicated that the direct effects of hypoxia on NTS were excitation, and the following inhibition was caused by the hypoxic damage to the neurons in the NTS. Therefore, elevated expression of FOS in NTS might be responsible for hypoxic respiratory depression. The exact functions of elevated expression of FOS in other neural nuclei of the brainstem after hypoxia remains a mystery, but TMP reduced FOS expression after hypoxia, which indicated at least that the protective effects of TMP against hypoxic respiratory depression are closely related to reducing FOS expression in the neural nuclei including NTS in the brainstem.

Glycolysis is induced by hypoxia and causes lactate production, which leads to extracellular acidification. Tissue acidification activates ASIC_1a_, which leads to inward sodium currents, and excess inward sodium currents lead to neuron swelling [25]. Meanwhile, ASIC_1a_ is also permeable to calcium ions, and calcium influx aggravates calcium overload, which leads to the necrosis of neurons. Previous literature demonstrated that ASIC_1a_ is the key player in hypoxic neuronal injury [26]. In the present study, expression of ASIC_1a_ increased in NTS and TZ of the brainstem after hypoxia. As discussed above, NTS plays an important role in hypoxic respiratory regulation. Smith et al. reported that there were some interacting links between the nucleus TZ and the ventral respiratory group, and they formed microcircuit to coregulate respiration [27]. Tian et al. showed that the electrical discharge of neurons in TZ was enhanced by microinjection of acidified artificial cerebrospinal fluid [28]. These studies indicated that TZ is also involved in the regulation of respiration. The present study showed that the expression of ASIC_1a_ increased in the nuclei NTS and TZ of the brainstem, and TMP attenuated the expression of ASIC_1a_ in the two nuclei induced by hypoxia. These findings indicated that the nuclei NTS and TZ were injured severely after hypoxia, the injury of the two nuclei may account for hypoxic respiratory depression, and the inhibitory effects of TMP on ASIC_1a_ expression might be another possible protective mechanism of TMP against hypoxic respiratory depression.

In the future, a lot of work can be performed to make our study richer and more in-depth. Does TMP antagonize hypoxic respiratory depression by reducing calcium overload and free radical damage? We can devote ourselves to the effects of TMP on the neuronal metabolism in the brainstem after hypoxia and the relationship with hypoxic respiratory reaction. Except the nucleus of NTS and TZ, what is the exact function of other FOS-positive neural nuclei in regulating respiration induced by hypoxia?

## 5. Conclusion

The present study identified that TMP pretreatment antagonized the respiratory depression in a rat model of acute hypoxia. TMP pretreatment significantly suppressed the upregulation of FOS in various nuclei in the brainstem induced by hypoxia, including AP, NTS, HN, LRN, IO, FN, and TZ. TMP pretreatment also attenuated the expression ASIC_1a_ in NTS and TZ induced by hypoxia. The protective effects of TMP on hypoxic respiratory depression might be related to its inhibition on the upregulation of FOS and ASIC_1a_ in the nuclei of the brain.

## Data Availability

The data used to support the findings of this study are included within the article.

## Supplementary Materials

Supplementary Materials Table S1. Effects of TMP on TI, TE, RF, Amp, and MBP at various timepoints after hypoxia treatment. Diaphragm EMG was recorded continuously, and the mean values of inspiratory duration (TI), expiratory duration (TE), respiratory frequency (RF), and inspiratory amplitude (Amp) were chosen as indexes to observe the changes of respiration. Before hypoxia treatment and 1 min, 10 min, 20 min, 30 min, and 40 min after hypoxia treatment were taken as our observation time points. ^*∗*^*P* < 0.05, ^*∗∗*^*P* < 0.01, *vs.* before hypoxia treatment in the same group.
