# Supplementary material for: Antagonistic Effects of Tetramethylpyrazine on Hypoxic Respiratory Depression in Rats
Source: Evid Based Complement Alternat Med. 2020 Sep 29;2020:6456017. doi: 10.1155/2020/6456017 (PMC7542524; doi:10.1155/2020/6456017)
Supplement: Supplementary Materials — Table S1. Effects of TMP on TI, TE, RF, Amp, and MBP at various timepoints after hypoxia treatment. Diaphragm EMG was recorded continuously, and the mean values of inspiratory duration (TI), expiratory duration (TE), respiratory frequency (RF), and inspiratory amplitude (Amp) were chosen as indexes to observe the changes of respiration. Before hypoxia treatment and 1 min, 10 min, 20 min, 30 min, and 40 min after hypoxia treatment were taken as our observation time points. ∗P < 0.05, ∗∗P < 0.01, vs. before hypoxia treatment in the same group. [file 6456017.f1.docx]

Table S1 Effects of TMP on TI, TE, RF, Amp and MBP at various time points after hypoxia treatment.

| Group | Time | TI (×10^-2^s) | | TE (×10^-2^s) | | RF (times/min) | | Amp (V. s) | MBP (mmHg) |
| --- | --- | --- | --- | --- | --- | --- | --- | --- | --- |
| TMP | Before-treatment | | 47.50±5.92 | | 69.75±21.75 | | 53±10 | 0.93±0.48 | 112±15 |
|  | 1min | | 46.60±4.76 | | 64.25±22.53 | | 56±9 | 0.95±0.50 | 109±13 |
|  | 10min | | 48.00±6.32 | | 71.50±20.82 | | 51±10 | 1.00±0.44 | 115±17 |
|  | 20min | | 47.50±6.40 | | 72.25±20.17 | | 51±9 | 1.03±0.47 | 110±16 |
|  | 30min | | 46.50±6.19 | | 73.25±20.99 | | 53±9 | 1.00±0.47 | 107±20 |
|  | 40min | | 47.80±7.72 | | 73.75±18.57 | | 52±8 | 1.00±0.49 | 110±18 |
| Hypoixa | Before-treatment | | 45.29±4.19 | | 58.80±8.00 | | 60±8 | 0.87±0.42 | 111±17 |
|  | 1min | | 37.43±3.78** | | 40.71±6.23* | | 79±9** | 1.13±0.30* | 54±13** |
|  | 10min | | 42.00±2.17* | | 50.28±5.11* | | 66±6* | 1.26±0.42** | 70±17** |
|  | 20min | | 41.57±2.34* | | 59.43±11.07 | | 60±7 | 0.89±0.44 | 64±6** |
|  | 30min | | 39.00±2.24* | | 78.85±9.79** | | 52±8* | 0.81±0.37 | 56±7** |
|  | 40min | | 37.57±2.29** | | 93.00±13.00** | | 46±8** | 0.59±0.21* | 55±8** |
| Hypoxia +TMP | Before-treatment | | 50.28±4.53 | | 67.57±19.95 | | 52±7 | 0.91±0.62 | 107±26 |
|  | 1min | | 43.71±5.09** | | 50.14±17.87** | | 67±10** | 1.37±0.71** | 69±21** |
|  | 10min | | 46.85±4.29* | | 52.29±15.71* | | 61±8** | 1.44±0.72** | 71±20** |
|  | 20min | | 46.71±4.19* | | 59.00±15.98* | | 57±9* | 1.39±0.70** | 68±14** |
|  | 30min | | 48.28±4.82 | | 67.71±18.45 | | 52±6 | 1.00±0.52 | 59±12** |
|  | 40min | | 48.57±5.38 | | 73.28±20.23 | | 49±6 | 0.90±0.58 | 58±10** |

**P*<0.05, ***P*<0.01, *vs* before hypoxia treatment in the same group. TI: Inspiratory duration, TE: expiratory duration, RF: respiratory frequency, Amp: inspiratory amplitude; MBP: mean blood pressure.
